# Development of a Briquetting Method for Dust from High-Carbon Ferrochrome (HC FeCr) Crushing Using Vibropressing on an Industrial Scale and Its Subsequent Remelting

**DOI:** 10.3390/ma18112608

**Published:** 2025-06-03

**Authors:** Otegen Sariyev, Maral Almagambetov, Nurzhan Nurgali, Gulnur Abikenova, Bauyrzhan Kelamanov, Dauren Yessengaliyev, Assylbek Abdirashit

**Affiliations:** 1 Department of Metallurgy and Mining, K. Zhubanov Aktobe Regional University, Aktobe 030000, Kazakhstan; bkelamanov@zhubanov.edu.kz (B.K.); dyessengaliyev@zhubanov.edu.kz (D.Y.); 2 ERG Research and Engineering Center, Astana 010000, Kazakhstan; maral.almagambetov@erg.kz (M.A.); nurzhan.nurgali@erg.kz (N.N.); gulnur.abikenova@erg.kz (G.A.)

**Keywords:** high-carbon ferrochrome (HCFeCr), briquetting, aspiration dust (AD), vibropress, binder, remelting, strength, gas cleaning dust and particle size distribution

## Abstract

The article provides a brief overview of technologies and methods for processing dispersed metallic waste generated during ferroalloy production, including high-carbon ferrochrome (HCFeCr). It is noted that the most cost-effective and rational method for reusing metallic dust is briquetting. Considering the development of briquetting technologies, as well as the latest equipment and binder materials involved in this process, aspiration dust from ferrochrome crushing can be fully utilized in metallurgical recycling. To verify this assumption, laboratory studies were conducted using polymer-based binders and liquid glass as a baseline option. The methodology of briquetting using both laboratory and industrial presses is described, along with an assessment of the mechanical properties of the briquettes. The studies indicate that the introduction of an inert filler (gas-cleaning dust) into the metallic dust composition improves the briquetting ability of the mixture by enhancing adhesion between metal particles and the binder. The obtained industrial briquette samples exhibit high mechanical strength, ensuring their further use in metallurgical processing. The study concludes that semi-dry briquetting using hydraulic vibropresses is a promising approach for the utilization of dispersed ferroalloy waste.

## 1. Introduction

One of the key stages in the final phases of high-carbon ferrochrome (HCFeCr) production is the crushing and screening of metal ingots. These processes are carried out in crushing and screening complexes (CSC) or production lines that include jaw crushers, screens, and conveyor systems [1]. The technological operations of crushing and fractionation inevitably result in the formation of certain amounts of fine fractions and dispersed metallic dust in addition to commercial-sized fractions. The latter is captured by a dry aspiration system, which is also an integral part of the CSC.

Screening fines are reused in metal casting technology as a bedding material, partially fusing with the ingot, or are sold to consumers at a discounted price. However, similar utilization of metallic dust is not feasible due to its high dispersity. More than 96% of the material consists of powder with a particle size of less than 0.071 mm, as clearly demonstrated in Table 1 below.

Currently, the metallic dust generated from HCFeCr crushing (hereinafter referred to as aspiration dust, or AD) is disposed of in industrial plants by remelting in ore-smelting furnaces to produce FeCr-600 and FeCr-650 grades using complex thermite technology. Several thousand tons of AD formed at domestic enterprises are remelted annually using this method [2,3,4].

**Figure 1:**
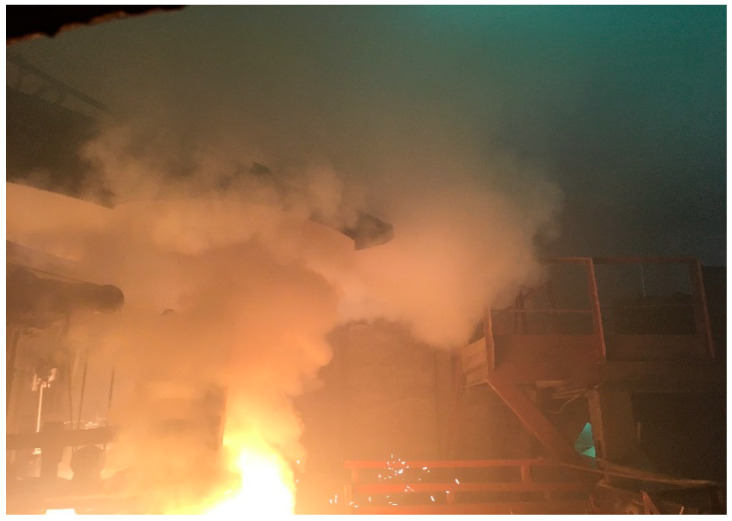
Carryover of aspiration dust (AD) from the furnace during remelting.

The main and significant drawback of the applied method is the high rate of mechanical losses of AD during furnace charging, as well as dust emissions from the furnace itself during the remelting process. This is clearly illustrated in Figure 1.

According to preliminary data, losses may amount to up to 15% of the annual volume of AD generated. Given this, there is a need to minimize or eliminate such losses through rational methods of recycling.

When aspiration dust (AD) is carried off, it primarily settles on the power supply components and electrode contact pads, as well as on the electrode lifting systems of the furnace. This leads to short circuits, damage to the power supply elements, and contributes to the burning through of the copper electrode contact pads. In addition, the presence of this dust causes jamming of the electrode lifting mechanisms, which can lead to their failure.

The primary and significant drawback of the current method is the high mechanical loss of aspiration dust (AD) during furnace charging, as well as dust carryover from the furnace itself during remelting. Literature sources describe various methods for processing dispersed ferroalloys, including their use in powder metallurgy [5], self-propagating high-temperature synthesis (SHS) methods [6,7,8], direct remelting, and for producing modifiers [9,10,11]. However, these methods have several disadvantages, such as complex technological operations and stringent requirements for the chemical composition of the final product.

For example, our earlier pilot-scale industrial tests on the injection of aspiration dust (AP) into liquid metal in a ladle using an injection unit were accompanied by significant heat losses and the formation of large amounts of metal crusts. Metal losses in the form of solidified deposits amounted to 30% of the molten metal mass. Taking this into account, further tests on injecting AP directly into the furnace melt were discontinued. The decision was based on the small volume of slag-metal melt formed in the furnace during smelting (6–7 tons, excluding the melt used to form the hearth lining), as well as the design of the furnace itself. The furnace used for loose AD remelting is an open-type furnace with a high heat loss coefficient. In the case of AP injection into the furnace, bath cooling would occur, requiring additional heat input and, consequently, increased energy consumption.

In this regard, the most rational approach for processing metallic dust is its preliminary agglomeration, followed by remelting in smelting furnaces [12,13]. Given the physical characteristics of AD—such as high dispersity, low wettability, and the sharp-edged shape of metal particles—the optimal agglomeration method in this case is briquetting [14].

One of the well-known methods for agglomerating fine-grained raw materials for subsequent metallurgical processing is the so-called cold briquetting. There are existing techniques for producing briquettes from fine ores and concentrates, metallurgical production waste, and carbon-containing dusts [15,16,17,18,19,20,21]. Using dispersed materials in agglomerated form, on the one hand, prevents their entrainment by off-gases, and on the other, ensures sufficient gas permeability of the charge column in metallurgical units. Therefore, agglomerated materials must possess sufficiently high cold and hot strength properties [22,23,24].

In other words, briquettes must retain their integrity at all stages of the metallurgical process. Each type of metallurgical raw material has its own technological features for briquette production, as well as specific types of binders. In turn, the choice of binder must consider the specific characteristics of the briquetting process depending on the nature of the solids. With proper binder selection, it is possible to ensure the required strength properties of briquettes both in the cold state and at metallurgical process temperatures. Moreover, the binder should not introduce harmful impurities into the final alloy or increase slag generation.

While inorganic binders such as cement and bentonite have long been used for agglomeration in ferrous metallurgy, polymer-based organic binders are currently gaining popularity and may potentially replace inorganic ones entirely [25,26].

Typically, polymer binders decompose at high temperatures without releasing hazardous decomposition products and completely volatilize.

Taking into account the experience of domestic enterprises in processing dusts from dry gas cleaning systems of ferroalloy furnaces—and the lack of industrial experience in processing metallic dust—the research focused on the agglomeration of aspiration dust (AD) generated during the crushing of high-carbon ferrochrome.

Briquetting was selected as the base processing technology, as it is the most accessible and economically justified method for the utilization of fine materials.

Unlike traditional approaches based on direct remelting or agglomeration using standard binders, this study proposes using dry gas cleaning baghouse dust (GCD) as a filler in the briquetting of aspiration dust.

Laboratory and industrial tests of the briquetting technology were carried out, aimed at adapting the existing briquetting equipment fleet at industrial facilities to new types of polymer-based binders, replacing conventionally used sodium silicate, cement, and bentonite.

The application of a modified mixture composition enabled the production of an industrial batch of briquettes that met mechanical and thermal strength requirements and confirmed the feasibility of their metallurgical processing into commercially acceptable ferrochrome by chemical composition at an industrial scale.

## 2. Materials and Methods

At the initial stage of laboratory experiments, various mixture compositions were prepared, selecting types of binders and inert materials to address cases of poor adhesion with aspiration dust (AD). Two materials were selected as binders, sodium liquid glass (LG) and a polymer binder based on lignosulfonate (polymer). The choice of liquid glass as a binder in amounts up to 6% (based on the dry weight of the briquetted material) was based on many years of experience at the Donskoy Ore-Dressing and Processing Plant in briquetting fine chromite ores [27]. Meanwhile, polymer-based binders have proven effective in the agglomeration of fine chrome-containing materials [28].

Combinations of both binders were also considered, based on literature data. According to sources [29,30], the addition of lignosulfonate can modify liquid glass by imparting it with higher binding properties. As an inert filler, dry gas cleaning dust (GCD) from ferroalloy furnaces was used. The chemical compositions of AD and GCD are presented in Table 2 and Table 3.

Next, all mixture variants for briquetting were consolidated into a single table. When compiling the mixture options presented in Table 4, the results of previously conducted laboratory and industrial experiments [27,31] were taken into account.

After dosing the components of the charge according to Table 4, dry mixing was carried out in an EIRICH EL1 mixer until the mixture was completely homogenized. The resulting mixture was then moistened, and mixing continued until a homogeneous consistency was achieved. The moisture content varied between 3% and 5% of the dry material weight. The finished mixture was poured into a mold (mold channel diameter—30 mm) and briquetted on a press model IP-1000-1 (Techpribor Plant, Moscow, Russia) with a compression force of 30 kN per briquette (with a calculated force of 425 kg per cm^2^). The resulting briquettes, 30 mm in diameter and 40 mm in height, were subjected to forced drying in a Nabertherm TR 420 (Nabertherm GmbH, Lilienthal, Germany) drying oven at a temperature of 120 °C for 3 h. After drying, the breaking strength of the briquettes was measured on a test press model RB-1000 (Techpribor Plant, Moscow, Russia). The strength of the raw briquettes was also measured separately. For strength measurements, 10 briquettes were selected from each batch and sequentially placed with the long axis perpendicular to the pressing punch of the test press, and the strength measurement process was carried out with an acceptable deviation of 3 kg for raw briquettes and 10 kg for dried briquettes.

**Figure 2:**
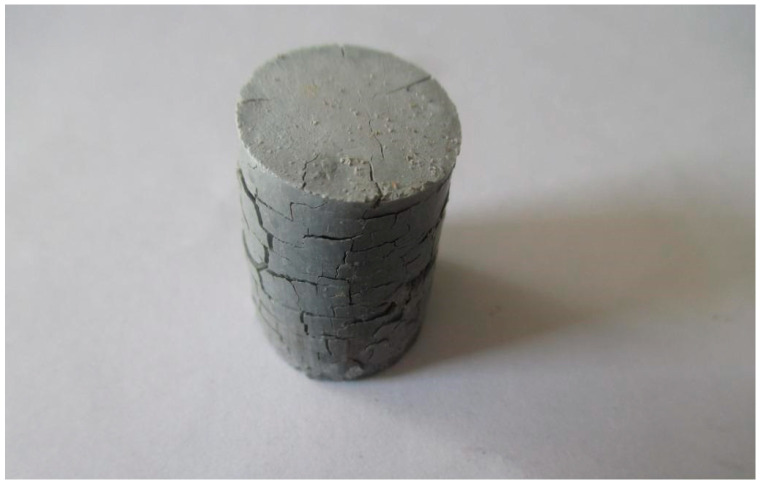
Appearance of Briquettes.

It should be noted that when briquetting the mixture with pure AD (variants No. 1–6), regardless of the binder type, the raw briquettes had extremely low strength and were easily destroyed. Increasing the compression force led to the formation of deep transverse cracks and ruptures, indicating over-pressing. This is clearly demonstrated in Figure 2.

The introduction of inert fillers in the form of gas-cleaning dust (GCD) did not stabilize the briquetting process in mixtures where liquid glass was used as a binder (mixtures No. 7–10). However, in the remaining mixtures, the addition of inert fillers had a positive effect (mixtures No. 11–18). The measured strength characteristics of the resulting briquettes for each mixture variant are presented in Table 5.

According to Table 3, briquettes from almost all mixture variants exhibit high mechanical strength, meeting the required 150 kg per briquette standard for briquetted chrome raw materials used in domestic enterprises. However, special attention should be given to briquettes with the highest green strength. Given that industrial briquetting equipment subjects raw briquettes to significant mechanical stress (such as screening and manual handling), mixtures with the highest green strength should be prioritized. The best-performing mixtures under these conditions are No. 13 and No. 14, which combine AD and GCD while using a polymer-based binder. The polymer binder used is widely applied in non-ferrous metallurgy, particularly in the agglomeration of copper-containing raw materials, and should not pose any technological or environmental difficulties [32,33]. They are widely used as a binder and plasticizer in the production of cast iron, steel, ore agglomeration, acid pickling, metal hardening, etc. Furthermore, due to the small amount of polymer binder used, it cannot have a significant impact on the final melting products of high-carbon ferrochrome (slag, metal).

Following the laboratory-scale briquetting results, test trials were conducted on industrial presses to produce full-scale industrial samples.

Briquetting was carried out using semi-dry pressing on an industrial hydraulic vibropress of the TITAN-Z3 model (TechnoMashStroy LLC, Smolensk, Russia). The press characteristics are presented in Table 6. Briquetting was performed according to the methodology described below without changing the compositions of the briquetted mixtures.

## 3. Results and Discussion

**Figure 3:**
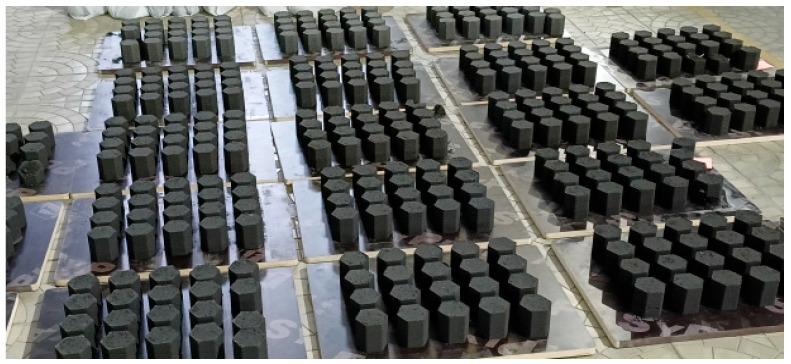
Appearance of the briquettes.

The briquetting materials and binder were weighed separately and then loaded into a single-screw mixer for dry mixing, which lasted 7 min. Water was then added, and wet mixing continued for approximately 7 min until the water was fully distributed throughout the mixture. Periodically, the mixer blades were cleaned of adhering material. After mixing was completed, the prepared mixture was extracted from the mixer and loaded into the receiving compartment (feeder) of the vibropress. The press was then activated to produce briquettes. The resulting briquettes had a uniform hexagonal shape, with each side measuring 40 mm in width and a briquette height of 80–83 mm. The appearance of the briquettes is shown in Figure 3.

**Figure 4:**
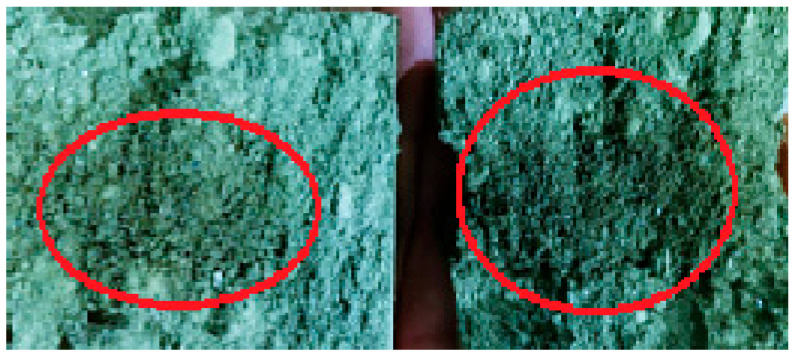
Briquette core after 3 h of drying.

The briquettes were subjected to forced drying at a temperature of 120 °C and also dried under natural conditions for three days. Initially, it was planned to dry the briquettes for three hours, as in laboratory experiments. However, after the specified drying time, the core of the briquette remained insufficiently dried (Figure 4). To eliminate this issue, the drying time was extended to five hours.

**Figure 5:**
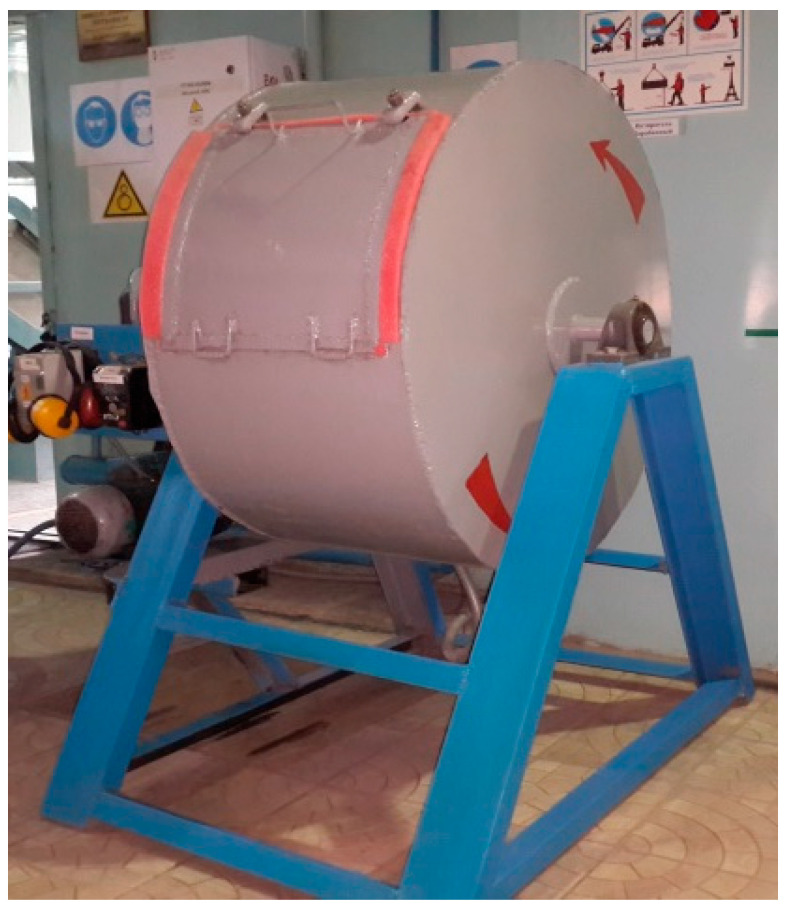
General view of the testing drum.

Upon completion of drying under both natural and forced conditions, measurements of the briquettes’ strength characteristics were conducted, including splitting, impact, drop resistance, and abrasion. The splitting strength of the briquettes was measured using a testing press model IP-50. The impact and abrasion strength measurements were carried out according to the methodology based on GOST 15137-77 [34] (Figure 5). The total iron content was determined following GOST 22772.4-77 [35].

The sample for testing is loaded into the drum, securely closed with a lid, and then set in motion. After completing 200 rotations, the drum is stopped, and the entire material is unloaded and sieved first through a sieve with a 5 mm mesh size. The fraction smaller than 5 mm is then further sieved through a sieve with a 0.5 mm mesh size. The obtained fractions—greater than 5 mm, between 5 mm and 0.5 mm, and smaller than 0.5 mm—are weighed separately.

The impact strength of the briquettes (X) in percentage is calculated using the following formula:

$$X=\frac{m_{1}}{m_{1}+m_{2}+m_{3}}\cdot 100$$

The abrasion resistance of the briquettes (X_1_) in percentage is calculated using the following formula:

$$X_{1}=\frac{m_{3}}{m_{1}+m_{2}+m_{3}}\cdot 100$$

where

m_1_—mass of the fraction larger than 5 mm after drum testing, kg;
m_2_—mass of the fraction between 5 mm and 0.5 mm after drum testing, kg;
m_3_—mass of the fraction smaller than 0.5 mm after drum testing, kg.

**Figure 6:**
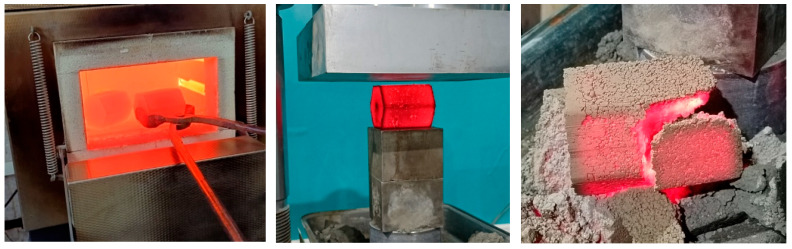
Measurement of the hot crushing strength of briquettes.

The next measurements were conducted to determine the hot strength of the briquettes (hot crushing strength). For this purpose, the muffle furnace Nabertherm TR 420 was heated to a temperature of 1000 °C. Once the target temperature was reached, the furnace door was opened, and the briquettes were placed inside. The door was then closed, and the holding process began. The briquettes remained in the furnace for approximately 15 min to allow for temperature equalization, as some heat is lost when the door is opened. After the holding time was completed, the briquettes were removed from the furnace and tested for crushing strength using a compression testing machine (Figure 6).

After completing all measurements of the briquettes’ strength characteristics (crushing, impact, drop, abrasion, and hot strength), the results were consolidated into Table 7. This table also includes the enterprise’s internal standard requirements for briquetted raw materials.

The obtained briquette samples from the AD and GCD mixture demonstrate sufficient crushing strength, exceeding the target values for this parameter. The mechanical strength indicators for drop resistance, impact, and abrasion—simulating the mechanical loads during transportation to the smelting units—also show high values, surpassing the target requirements.

The hot strength of the briquettes ranges from 32 to 45 kg/briquette, compared to the target value of 40 kg/briquette.

Overall, the conducted experiments demonstrated the feasibility of briquetting ASP, provided that an inert material such as PSG is added to the mixture. The amount of PSG introduced at a ratio of 20:80 and a binder consumption of 3% (of the dry mass of ASP and PSG) ensures the production of briquettes with high strength characteristics, sufficient for further metallurgical processing. Increasing the binder consumption to 4% does improve the strength characteristics of the briquettes, but it leads to higher costs due to the binder price and also results in a lower hot strength index.

**Figure 7:**
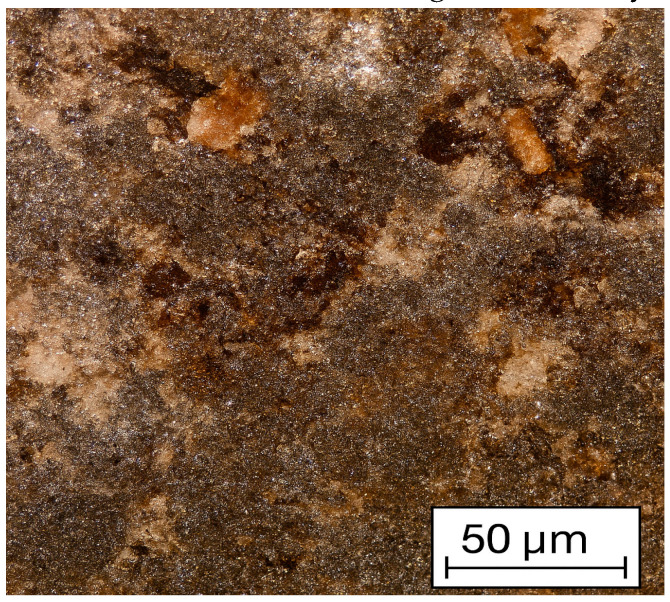
Microphotograph of the structure of a chrome ore briquette under natural light [34].

Due to the low wettability (adhesion to the binder) of ASP particles, the addition of PSG serves as a kind of shell-filler. Due to its dispersion (87% of the fraction is −0.02 mm compared to 96% of the fraction being −0.071 mm for ASP) and low bulk density (0.2 t/m^3^ compared to 2–4 t/m^3^ for ASP), it allows for almost complete encapsulation of the metal particles. The addition of the polymer binder leads to the formation of adhesive films on the surfaces of PSG particles, which, in turn, hold the ASP particles together in the form of a framework. This statement aligns well with the work [36], where fine chrome ore briquetting was conducted. The mentioned work states that the polymer forms a film on the surface of the chrome ore grains, thereby creating a thin adhesive surface (Figure 7).

Considering that PSG is a dispersed chrome ore captured by the aspiration system, it is most likely that this hardening mechanism is at work in the briquettes made from a mixture of AD and PSG. The high strength characteristics of the briquettes, including enhanced resistance to impact, abrasion, and hot strength, prevent the dispersion of AD particles, as seen in their smelting in bulk. This, in turn, will contribute to a more complete involvement of AD in the smelting process with minimal losses.

Next, based on the obtained results, a pilot batch of briquettes weighing 63 tons (dry weight) was produced according to option No. 13 with the aim of conducting industrial tests for their smelting. The pilot-industrial tests for the smelting of briquettes made from a mixture of AD and PSG were carried out on an industrial open-type shaft furnace with a transformer power of 16 MVA.

Typically, in the smelting of ferrochrome grades FX 600-650 using complex thermoreduction, several types of metal concentrates from HC FeCr production, as well as LC-MCFeCr metal concentrate, are used. To minimize errors in evaluating technical and economic Indicators (TEI), it was decided to use only one type of metal concentrate (from HC FeCr).

According to the test program, three days before the start of briquette smelting, the furnace was switched to the following charge composition:

- AD (bulk)—1 ton;
- Metal concentrate fraction 0–5 mm (Cr content not less than 45%)—8 tons;
- Chromite ore—1 ton;
- Ferrosilicochrome (FeSiCr)—0.4 tons;
- Lime—0.8 tons.

The chemical composition data of the charge materials used during the industrial tests are presented in Table 8.

The above charge composition was adopted as the baseline variant and was processed over three days, during which 27 smelts were conducted. The charging of AP into the furnace was accompanied by intense dust formation, complicating its uniform distribution in the furnace bath. Additionally, dust emissions into the workshop atmosphere were observed, bypassing the gas cleaning system.

Furthermore, periodic ejections of AD from the furnace were noted during its smelting, along with overheating of the taphole areas and furnace pockets. The tapping and casting of the melt were carried out in accordance with the established regulations for smelting processes in the specified furnace. Metal and slag samples were taken using a ladle sampling method. After each tap, the weight of the produced slag and metal ingots was measured without fail.

After completing the baseline period, briquettes were introduced as a replacement for bulk AD. During the experimental period, the furnace operated with the following charge composition:

- Briquettes AD—1.3 tons
- Metal concentrate fraction 0–5 mm (Cr content not less than 45%)—8 tons
- Chromite ore—0.9 tons
- FeSiCr—0.4 tons
- Lime—0.8 tons

During the experimental period, 48 smelts were conducted over six days. The furnace personnel noted the absence of dust formation and the convenience of briquette charging, which allowed for uniform material distribution across the entire furnace bath.

Additionally, during the experimental period, AD ejections during smelting ceased, and overheating of the furnace pockets and taphole areas was no longer observed. All technological operations related to smelting and casting were carried out in accordance with the established regulations for the remelting process.

The technical and economic indicators of the baseline and experimental periods are presented in Table 9.

During the experimental period, 48 smelts were conducted over 6 days. The furnace personnel noted the absence of dusting and the ease of loading briquettes, which allowed for an even distribution of the material throughout the furnace bath. Briquettes were mainly loaded around the perimeter of the furnace, which allowed for a uniform drop into the forming liquid melt. In this case, the briquettes prevented the formation of slag rings on the bath perimeter, while their mass helped them sink well into the liquid melt near the arc combustion zone. This loading method compensated for the absence of a well-developed surface of ferrochrome in the briquettes and increased the melt’s absorption of AD (from the briquette composition).

During the experimental period, AD emissions during the smelting process ceased, as well as heating of the furnace pockets and tuyères. All technological operations for melting and pouring were carried out according to the existing regulations for smelting processes. Additionally, during the industrial trial period using briquettes, the temperature of the furnace lining, the temperature of the exhaust gases, and the thickness of the furnace slag after pouring were monitored. In comparison with the baseline period, when using briquettes, the furnace lining temperature and exhaust gas temperature remained at the same level, while the slag thickness decreased on average by 80 mm. Furthermore, the freezing of the furnace bottom ceased, with an increase in its depth, indicating that the melt temperature was sufficient.

**Figure 8:**
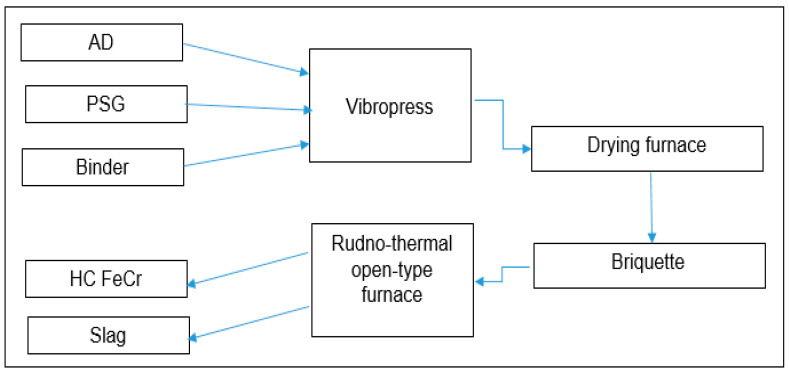
Block diagram of the process during the EPI period.

The block diagram of the Experimental Industrial Testing (EPI) period with briquettes is shown in Figure 8.

The technical and economic indicators of the baseline and experimental periods are presented in Table 10.

The results of industrial trials show that during the test period, there was an increase in the specific volume of metal produced. The specific reduction in metallic chromium losses with dust decreased from 10 kg/t under the baseline mode to 0.58 kg/t during the test period. At the same time, a reduction in the specific consumption of electric power by 364 kWh/t (Cr) and electrodes by 0.13 kg/t (Cr) was observed.

The costs of briquetting, taking into account the price of the binder, energy resources, and auxiliary materials, were preliminarily estimated at 30 USD. The additional amount of chromium obtained due to the use of briquettes amounted to 0.491 tons per heat. Thus, given the baseline smelting cost of AD at 112 USD and the selling price of HCFeCr at 2600 USD/t Cr, the economic benefit reached 1135 USD per heat.

## 4. Conclusions

Agglomeration of AD in its pure form by briquetting is not feasible due to the specific grain morphology and low wettability. The use of filler material in the form of dust from baghouse filters of dry gas cleaning systems in HCFeCr smelting furnaces enhances the interparticle adhesion of AD, ultimately enabling its agglomeration through briquetting.

Moreover, baghouse filter dust serves as an additional source of chromium and a slag-forming component, allowing for the exclusion of recycled slags during AP remelting.

The results of laboratory studies on briquetting were confirmed under industrial conditions. It was found that pressing a mixture of aspiration dust and bag filter dust in an 80:20 ratio using 3% binder (based on the dry weight of both aspiration dust and bag filter dust) through semi-dry pressing on hydraulic presses allows for the production of industrial briquette samples with high strength characteristics.

The conducted research confirmed that briquettes produced using the developed technology exhibit sufficient resistance to splitting, impact, drop, and abrasion, while also maintaining their strength when heated up to 1000 °C. These characteristics allow their use in smelting units without compromising quality.

Industrial trials of HCFeCr smelting using briquettes made from an AD and GCD mixture were conducted, with an assessment of technical and economic indicators (TEI) compared to bulk AD smelting. The following results were achieved:

- Increase in the yield of marketable metal by 16%;
- Reduction in metallic chromium losses with dust from the gas cleaning system from 10 kg/t to 0.58 kg/t;
- Reduction in energy consumption by 13.9%;
- Reduction in electrode consumption by 5.7%.
- Reduction in slag ratio by 13%;
- Reduction in chromite ore consumption by 10%.

Preliminary economic calculations show savings of 1135 USD per heat, confirming the feasibility of using aspiration dust briquetting without increasing the cost of HCFeCr production.

## Figures and Tables

**Table 1:** Fractional Composition of Dust from HCFeCr Crushing.

Fraction, mm	Content, %
+0.2	0.07
−0.2 + 0.16	0.14
−0.16 + 0.125	0.31
−0.125 − 0.071	2.75
−0.071 + 0.0	96.73

**Table 2:** Chemical composition of aspiration dust (AD), %.

Cr	Si	C	S	P	Fe
64.89	2.54	8.24	0.027	0.014	other

**Table 3:** Chemical composition of dry gas cleaning dust (DCBD), %.

MgO	SiO_2_	CaO	Cr_tot_	Al_2_O_3_	Fe_tot_	C
32.8	18.0	0.4	14.9	6.8	4.2	5.8

**Table 4:** Compositions of mixtures for briquetting.

Option Number of the Mixtures	Materials	Type of Binder *	Binder Consumption, %
1	AD	LG	6
2	AD	LG	8
3	AD	Polymer	3
4	AD	Polymer	4
5	AD	LG/Polymer	3:3 *
6	AD	LG/Polymer	4:4
7	AD + GCD (90/10)	LG	6
8	AD + GCD (90/10)	LG	8
9	AD + GCD (80/20)	LG	6
10	AD + GCD (80/20)	LG	8
11	AD + GCD (90/10)	Polymer	3
12	AD + GCD (90/10)	Polymer	4
13	AD + GCD (80/20)	Polymer	3
14	AD + GCD (80/20)	Polymer	4
15	AD + GCD (90/10)	LG/Polymer	3
16	AD + GCD (90/10)	LG/Polymer	4
17	AD + GCD (80/20)	LG + Polymer	3:3
18	AD + GCD (80/20)	LG + Lignosulfonate	4:4

* (LG consumption 3%: Polymer consumption 3%).

**Table 5:** Breaking strength results of briquettes.

No. of Mixture Options	Composition	Binder, %		W, %	Green Splitting Strength, kg/Briquette	Splitting Strength After Drying (120 °C, 3 h), kg/Briquette
		Polymer	LG			
11	AD + GCD (90/10)	3	0	5	17	170
12	AD + GCD (90/10)	4	0	5	28	188
13	AD + GCD (80/20)	3	0	5	37	311
14	AD + GCD (80/20)	4	0	5	41	405
15	AD + GCD (90/10)	3	3	3	27	182
16	AD + GCD (90/10)	4	4	3	22	232
17	AD + GCD (80/20)	3	3	4	16	149
18	AD + GCD (80/20)	4	4	4	21	254

**Table 6:** Technical Specifications of the TITAN-Z3 Vibropress.

Parameter Names	Value
Briquette Production Capacity (80 × 80 mm), pcs/h	500
Molding Zone Dimensions, mm × mm	400 × 600
Briquette Shape	Hexagonal
Forming Cycle, s	30–60
Hydraulic System Pressure, MPa	6–12
Molding Zone Height, mm	50–200
Vibration Table Frequency, Hz	45–85

**Table 7:** Strength Characteristics of Briquettes.

Indicators	Varinat No. 13	Varinat No. 14	Target Values
Splitting Strength, kg/briquette	177/205 *	189/263	150
Impact Strength, %	87/89	91/91	85
Abrasion Resistance, %	14/12	12/11	15
Drop Strength, %	93/96	96/98	90
Hot Strength, kg/briquette	45	32	40

* Note—Natural Drying/Forced Drying.

**Table 8:** Chemical Composition of Charge Materials, %.

Material	Cr	Cr _met_	Cr_2_O_3_	Si	SiO_2_	CaO	MgO	Al_2_O_3_	FeO	C	S	P
**Metal concentrate**	48.27	45.07	-	6.52	-	-	-	-	-	-	-	0.029
**Chromite ore**	-	-	50.75	-	7.2	0.88	11.61	3.74	17.55	6.91	0.36	0.007
**AD**	56	51.17	-	-	-	-	-	-	-	-	-	-
**Briquette**	-	42.48	-	-	-	-	-	-	-	-	-	-
**Lime**	-	-	-	-	-	91.32	-	-	-	-	-	0.004
**FeSiCr**	32.32	-	-	44.05	-	-	-	-	-	0.03	-	-

**Table 9:** Technical and Economic Indicators (TEI) of HCFeCr Smelting in the Furnace Using AD.

No.	Parameters	Experimental and Industrial Trial Periods	
1	Charge input, kg	Baseline	Experimental
	Metal concentrate	213,000	374,000
	AD	30,340	-
	Briquettes AD	-	61,937
	Chromite ore	27,200	44,000
	FeSiCr	10,800	19,200
	Lime	21,600	41,600
2	Produced metal, physical weight, kg	120,040	246,370
3	Produced slag, physical weight, kg	97,732	174,766
4	Slag ratio	0.81	0.71
5	Metal composition, %		
	Cr	67.69	68.24
	Si	0.68	0.68
	C	6.69	6.71
	S	0.02	0.03
	P	0.03	0.03
6	Average melt weight of Cr_met_, kg	3012	3503
7	Dust collection system output, kg	4220	3960
8	Cr_met_ in dust collection system, kg	1208	144
9	Specific power consumption, kWh/ton (Cr)	2732	2368
10	Specific electrode consumption, kg/t (Cr)	6.44	6.12

**Table 10:** Technical and Economic Indicators of Ferrochrome Production on the Furnace Using AD.

No.	Parameters	Periods of Technological Operations for Melting	
1	Charge composition specified, kg	Baseline	Experimental
	Metal concentrate FeCr	213,000	374,000
	Aspiration dust in loose form	30,340	-
	Briquettes	-	61,937
	Cr ore	27,200	44,000
	FeSiCr	10,800	19,200
	Lime	21,600	41,600
2	Metal output, kg	120,040	246,370
3	Slag output, kg	97,732	174,766
4	Slag ratio	0.81	0.71
5	Metal composition, %		
	Cr	67.69	68.24
	Si	0.68	0.68
	C	6.69	6.71
	S	0.02	0.03
	P	0.03	0.03
6	Average weight of Cr _metal_ per heat, kg	3012	3503
7	Volume of dust, kg	4220	3960
8	Cr_met_ of dust, kg	1208	144
9	Specific electricity consumption, kWh/ton (Cr)	2732	2368
10	Specific electrode consumption, kg/ton (Cr)	6.44	6.12

## Data Availability

The original contributions presented in this study are included in the article. Further inquiries can be directed to the corresponding authors.
